# Sweet Surprises: Decoding Tumor-Associated Glycosylation in Cancer Progression and Therapeutic Potential

**DOI:** 10.3390/cells15030233

**Published:** 2026-01-26

**Authors:** Eileena F. Giurini, Sam G. Pappas, Kajal H. Gupta

**Affiliations:** 1 Division of Pediatric Oncology, Department of Surgery, RUSH University Medical Center, Chicago, IL 60612, USA; 2 Division of Surgical Oncology, Department of Surgery, RUSH University Medical Center, Chicago, IL 60612, USA; 3 Department of Internal Medicine, University of Texas Medical Branch, Galveston, TX 77555, USA

**Keywords:** cancer immunotherapy, tumor microenvironment, cancer glycobiology, sialylation and Siglec signaling, glycan-targeted cancer therapy

## Abstract

Tumor-associated glycosylation is a defining hallmark of cancer, exerting profound effects on multiple aspects of tumor biology. This phenomenon arises from the central role of glycosylation in a wide range of cellular processes and its inherently diverse structural complexity. In cancer cells, aberrant glycosylation often results in the modification of glycoconjugate structures, leading to alterations in cell surface architecture that disrupt cellular homeostasis and signaling pathways. These changes can enhance tumor cell proliferation, invasion, and metastasis by modulating cell adhesion, receptor activation, and intracellular communication. Beyond its direct impact on cancer cells, tumor-associated glycosylation plays a pivotal role in shaping the tumor microenvironment. Aberrant glycan structures influence immune cell infiltration by altering antigen presentation and immune checkpoint interactions, contributing to immune evasion. Additionally, these modifications regulate angiogenesis by affecting endothelial cell function and promoting the formation of aberrant vasculature, which supports tumor growth and metastasis. Glycosylation also mediates tumor–stroma interactions, influencing extracellular matrix remodeling and fibroblast activation, further enhancing cancer progression. This interplay between cancer-associated glycan modifications and their functional roles in tumorigenesis presents a promising therapeutic approach. Unlike conventional treatments, glycan-targeting therapies confer high tumor specificity, operate independently of canonical immune checkpoint targets, and help mitigate immune cell exhaustion. This review explores commonly dysregulated glycan motifs implicated in tumorigenesis and delves into their mechanistic contributions to cancer pathogenesis. We then highlight emerging opportunities for therapeutic intervention, including the development of glycan-targeted therapies and biomarker-driven strategies for cancer diagnosis and treatment. We also outline where glycan-targeted agents (e.g., desialylating biologics, glycomimetics, and anti-glycan mAbs) can complement checkpoint blockade and potentially overcome immune escape.

## 1. Introduction

Cancer remains a leading cause of mortality, largely due to the limited efficacy of conventional treatments such as chemotherapy and radiotherapy, while aberrant protein glycosylation, a hallmark of cancer, actively contributes to tumor development and progression through specific glycan modifications. Nearly 2% of the human genome is dedicated solely to glycan-modifying genes that have been conserved in the genome for millions of years [1]. Glycan structures exhibit remarkable diversity and dynamism, which largely stems from their non-template-driven synthesis [2]. As essential components of glycosylation—the enzymatic process of attaching carbohydrates to macromolecules—glycans contribute to the formation of glycoconjugates, including glycoproteins and glycosphingolipids. These modifications are highly prevalent, and nearly half of the mammalian proteome undergoes glycosylation [3]. Glycosylation primarily occurs within the endoplasmic reticulum and Golgi apparatus, and glycoconjugates are predominantly found on the cell surface or are integrated into the extracellular matrix (ECM). Their strategic localization enables glycans to mediate critical cellular interactions, including cell–cell and cell–molecule communication. These interactions influence a range of essential processes such as intracellular signaling, receptor activation, gene transcription, and molecular trafficking. Given the fundamental role of glycosylation in maintaining cellular homeostasis, it is unsurprising that disruption of glycan function can lead to significant pathological consequences.

Aberrant glycosylation has been shown to be embryonically lethal in many instances, highlighting its essential role in maintaining cellular processes compatible with life [2]. The spectrum of pathologies associated with disruptions in glycosylation is immense. While some congenital disorders of glycosylation (CDG) are compatible with survival, defects even in a single enzymatic step of the glycosylation pathway can result in profound neurological and musculoskeletal abnormalities. Aberrant N-glycosylation on proteins in the brain has also been linked to neurodegenerative diseases prevalent later in life, such as Alzheimer’s Disease and Parkinson’s Disease [1,2]. Glycans and glycosylation also play pivotal roles in infectious diseases. Numerous pathogens exploit host-expressed glycans as adhesion receptors, which are then co-opted to facilitate microbial colonization and cellular invasion. Additionally, bacterial toxins such as tetanus toxin from *Clostridium tetani* and botulinum toxin from *Clostridium botulinum* bind to membrane-associated glycans to mediate intracellular translocation into host cells. Though playing an integral role in mediating infectious diseases, glycans also contribute significantly to attenuating the progression of these diseases, in addition to overall modulation of host immunity. Glycan structures are integral to the trafficking of leukocytes to infected tissues through the modulation of endothelial adhesion structures. Differentiation, activation, and cell survival of T and B cells are regulated through modifying the glycosylation of glycoproteins CD43 and CD45 [4]. Given the vast reach of glycan structures in perpetuating many distinct pathologies, it is unsurprising that this reach also extends to tumorigenesis.

Glycosylation aberrations are heavily implicated in malignant cell development, contributing to nearly all facets of cancer pathogenesis. As a result, altered glycosylation is now appreciated as a standalone hallmark of cancer [5,6,7]. A consequence of the advances in glycan profiling is the identification of a neoplastic glycan “signature,” or glycan structures that are enriched on malignant cells but not present on non-transformed counterparts. The following sections will comprehensively examine how cancer-associated alterations in glycan structures, otherwise known as tumor-associated glycosylation (TAG) structures, influence tumor cell behavior and contribute to the acquisition of cancer hallmark capabilities. These include sustained proliferative signaling, resistance to apoptosis, immune evasion, enhanced motility and invasiveness, and adaptation to the tumor microenvironment. By delineating the mechanistic roles of specific glycosylation patterns—such as branched N-glycans, sialylation, fucosylation, and truncated O-glycans—this review aims to elucidate the multifaceted contributions of glycan dysregulation to cancer pathogenesis and progression.

## 2. Tumor-Associated Glycoconjugates

**Figure 1:**
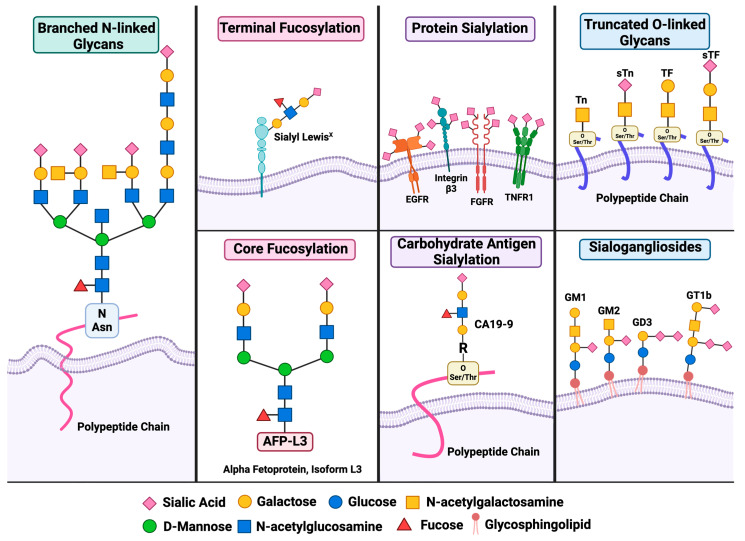
Common motifs of tumor-associated glycosylation include branched N-linked glycans, which are often elongated with galactose–N-acetylglucosamine repeats, and are abundant in cancer cells. Fucosylation, especially terminal fucosylation, generates structures like sialyl Lewis^X^, promoting tumor migration. Core fucosylation, as seen in the L3 isoform of alpha-fetoprotein, serves as a tumor biomarker. Sialylation of membrane receptors modulates intracellular signaling pathways that support tumor survival and metastasis. CA19-9, a sialylated O-glycan, has been studied as a biomarker and supports PDAC progression. Cancer cells also often express truncated O-glycans (e.g., T/TF antigens) due to altered glycosyltransferase activity, correlating with poor prognosis. Lastly, sialogangliosides on the membrane regulate signaling and cell–cell interactions, enhancing resistance to apoptosis and tumor growth. Created with BioRender Giurini, et. al. (2026) https://app.biorender.com/illustrations/69767be256ce86a62da689dc?slideId=4161238e-7491-4b29-8f0b-a2ec5ed01959.

A consequence of the advances in glycan profiling is the development of a neoplastic glycan “signature,”—a distinct and reproducible pattern of glycan structures that are specifically enriched or uniquely expressed on the surface of cancer cells, but are rare or absent in normal, non-transformed cells [8,9]. These glycan alterations arise due to the dysregulation of glycosylation enzymes, such as glycosyltransferases and glycosidases, during malignant transformation and tumor progression. The resulting glycan profiles serve as molecular fingerprints of cancer and are often associated with tumor subtype, stage, or aggressiveness [9]. The subsections below describe each of these glycoconjugates and their role in tumorigenesis. A graphical depiction of the molecular structure for each glycoconjugate has been provided in Figure 1.

Importantly, tumor-associated glycosylation is highly cancer-type specific. For example, pancreatic ductal adenocarcinoma is characterized by CA19-9 expression and hypersialylation driven by oncogenic KRAS–ST6GAL1 signaling [10,11], hepatocellular carcinoma exhibits diagnostic enrichment of core-fucosylated AFP-L3 [12,13], breast cancers frequently display increased branched N-glycans and ST6GAL1-associated epithelial–mesenchymal transition [5], whereas hematologic malignancies often exploit truncated O-glycans and Siglec-targetable antigens such as CD22 to support immune evasion and therapeutic resistance [14].

### 2.1. Branched N-Linked Glycosylation

N-linked glycosylation is a fundamental post-translational modification in which an oligosaccharide containing N-acetylglucosamine (GlcNAc) is covalently attached to the nitrogen atom of an asparagine (Asn) residue within a protein. This process begins in the endoplasmic reticulum and continues in the Golgi apparatus, where the glycan structure undergoes further development. N-glycans play a crucial role in various intracellular functions, including proper protein folding, quality control, and stabilization of protein half-life. They are also essential for modulating intracellular signaling pathways and mediating cell–cell and cell–matrix adhesion [15]. Because N-glycans are involved in such critical biological processes, even subtle alterations in their structure can have profound consequences for cellular function and integrity. One well-characterized cancer-associated modification is the branching of N-glycan structures. Branched N-glycans comprise two or more “arms” typically with a GlcNAc base, followed by galactose or poly-N-acetyllactosamine residues that elongate the branch [5,16]. These structures are often overexpressed on the surface of cancer cells due to dysregulated expression and activity of cancer-associated glycosyltransferases such as N-acetylglucosaminyl transferase V (GnT-V) [17].

In normal cells, complex N-glycans are predominantly bi-antennary, whereas malignant transformation is frequently associated with the emergence of tri- and tetra-antennary N-glycan structures driven by increased activity of MGAT5 (GnT-V), which promotes receptor clustering and oncogenic signaling [5,16,18]. In contrast, the addition of a bisecting N-acetylglucosamine by MGAT3 (GnT-III) sterically restricts further branching, disrupts galectin-mediated receptor lattice formation, and is generally associated with reduced growth factor signaling and suppression of tumor progression [19,20].

Branched N-glycans have been strongly implicated in tumor progression. For instance, metastatic tumors often express more highly branched N-glycans than their nonmalignant counterparts, suggesting a role in enhancing cancer cell motility and invasion [21,22]. These structures may facilitate detachment from the primary tumor and promote interactions with the ECM. These linkages impart a negative charge and support dissemination through the bloodstream [23]. In support of this, branched N-glycans on the vascular endothelial growth factor receptor-2 (VEGFR2) have been shown to strengthen its interaction with endothelial cells, enhancing pro-angiogenic signaling [24]. This increased VEGFR2 activation is thought to promote tumor vascularization and aid in cancer cell entry into systemic circulation [22].

### 2.2. Fucosylation

Fucosylation refers to the enzymatic addition of the monosaccharide fucose to proteins, glycoproteins, or glycolipids. This modification is orchestrated by a family of enzymes known as fucosyltransferases (FUTs), primarily within the Golgi apparatus. Fucosyltransferases give rise two fucose-based glycan moieties: terminal and core fucosylated structures [5]. Terminal fucosylation is catalyzed by FUT1 and FUT2 α-1,2 and α-1,3/4 FUTs [25]. Terminal fucosylation gives rise to the Lewis blood group antigen, sialyl Lewis^X^ (sLeX). sLeX is a terminal fucosylated tetrasaccharide, a prominent glycan structure that is associated with aggressive tumor behavior. Elevated sLeX expression has been linked to reduced disease-free survival, resistance to chemotherapy, and enhanced metastasis [26]. sLeX expression has been frequently correlated with poor clinical outcomes across multiple cancer types [27]. Selectins belong to the C-type lectin family, which are adhesion molecules expressed on endothelial cells. It is through selectin interaction that sLeX supports tumor cell migration and subsequent metastasis [28,29].

Core fucosylated glycans are also clinically significant in cancer detection. This process is mediated by α1,6-fucosyltransferases (FUT8), which add a fucose residue to the innermost N-acetylglucosamine (GlcNAc) of an N-glycan structure [30,31]. One of the most well-characterized examples of core fucosylation in cancer is found in alpha-fetoprotein (AFP), a glycoprotein primarily produced by the fetal liver and re-expressed in certain liver pathologies [12]. A core fucosyl residue present on AFP is essential to hepatocellular carcinoma (HCC) diagnosis. HCC, hepatitis B, and liver cirrhosis alike all present with elevated serum AFP [13]. However, the fucosylated L3 isoform of AFP (AFP-L3) is present only in HCC, distinguishing HCC from other liver pathologies. Thus, AFP-L3 is now being regarded as a clinically relevant tumor biomarker for HCC.

### 2.3. Sialylation

Sialylation, a specific and well-defined modification within the broader glycosylation landscape, plays a critical role in promoting cancer progression. This process involves the enzymatic addition of sialic acid residues to glycoproteins and glycolipids, primarily occurring in the Golgi apparatus. It is mediated by a family of membrane-bound sialyltransferases, which catalyze the formation of glycosidic linkages between sialic acid and the terminal positions of glycan chains. Distinct sialyltransferases catalyze the formation of specific sialic acid linkages on glycoconjugates. α2,3-linked sialylation is primarily mediated by the ST3GAL family, α2,6-linked sialylation by ST6GAL1 and ST6GAL2, and α2,8-linked sialylation by members of the ST8SIA family, which generate polysialic acid structures with important roles in tumor progression and immune modulation [32]. Increased sialylation of membrane-bound receptors such as integrins, EGFR, and FGFR1 can enhance their stability, clustering, and activation, leading to sustained signaling through pathways like PI3K/Akt, MAPK/ERK, and FAK/Src [33]. Functionally, receptor hypersialylation stabilizes/clusters RTKs and integrins and amplifies PI3K/Akt and MAPK/ERK signaling, promoting proliferation, survival, and motility [34,35]. These sialylated structures are frequently upregulated in cancer and contribute to various malignant phenotypes, including enhanced cell survival, immune evasion, and metastatic potential [11,33]. The sialyltransferases establish a glycosidic linkage between the C2 of the sialic acid donor and the C3, C6, or C8 hydroxyl of a glycoconjugate structure, forming α-2,3, α-2,6, or α-2,8 linkages, respectively [22]. On the cell membrane, the presence of sialic acid residues confers a negative charge to glycoconjugates via their carboxyl groups, shaping cell–cell and cell–immune interactions. that creates an electrostatic repulsion between cells, consequently promoting unobstructed cellular transit through the circulation [36]. In addition, sialic acids modulate cell–cell interactions, immune responses, intracellular signaling, and stem cell pluripotency [11]. Disruption of these regulatory processes contributes to tumor initiation and progression, and alterations in key mediators such as sialic acids can exert profound pathological effects.

In cancer cells, altered sialylation often manifests as elevated overall levels of sialic acid residues on the cell surface, often referred to as hypersialylation. This increase is understood to be a result of upregulated sialyltransferases, enzymes responsible for transferring sialic acid residues to other molecules. Notably, oncogenic mutations such as mutant KRAS have been shown to enhance sialyltransferase expression, which may contribute to the pronounced sialylation observed in KRAS-driven tumors, including those of the colon, lung, and pancreas [37]. Hypersialylation of tumor cells most often presents with overexpression of α-2,3 and α-2,6 linkages [11,33]. Increased tumor sialylation can also be attributed to ganglioside overexpression. Gangliosides are a subclass of glycosphingolipids that reside within the plasma membrane and are characterized by the presence of one or more terminal sialic acid residues [5]. Regardless of the specific glycan structure involved, elevated levels of sialylation on tumor cells are almost universally associated with unfavorable clinical outcomes and reduced overall prognosis [38].

Similarly to fucosylated glycans, sialylated glycans also have discrete roles in cancer detection and progression. Carbohydrate antigen 19-9 (CA19-9), is the sialylated form of tetrasaccharide Lewis antigen A, thus, alternatively known as sialyl Lewis^A^ [39]. CA19-9 is often elevated in the serum of patients with colorectal, gastric, esophageal, and ovarian cancers. Among various malignancies, CA19-9 demonstrates its greatest diagnostic accuracy—both in terms of sensitivity and specificity—in pancreatic ductal adenocarcinoma (PDAC). In this context, elevated serum levels of CA19-9 have been closely associated with disease burden, positioning its use as a prognostic indicator and potential marker for disease recurrence [40]. However, its usage as a biomarker has encountered some significant challenges, such as false positive results (elevated serum CA19-9) in nonmalignant pancreatic diseases [40]. CA19-9 is also functionally implicated in PDAC pathogenesis. In cooperation with KRAS^G12D^, CA19-9 mediated hyperactivation of epidermal growth factor receptor (EGFR) that perpetuated PDAC aggression in mice. In addition, CA19-9 expression was identified to promote PDAC metastasis in mice [10]. Additional studies support CA19-9 as a primary mediator of PDAC metastasis, specifically through direct interaction with E-selectin expressed on the endothelium [41].

Although individual glycan modifications are often discussed separately, multiple tumor-associated glycosylation changes converge on shared biological outcomes, including stabilization of oncogenic receptor signaling, suppression of anti-tumor immunity, and promotion of metastatic behavior. Across distinct glycan motifs, including branched N-glycans, fucosylation, and sialylation, structural remodeling consistently translates into common functional outcomes such as enhanced receptor clustering, activation of oncogenic signaling pathways, and modulation of immune cell interactions, as discussed in subsequent sections.

## 3. Functional Role of Glycans in Tumorigenesis

**Figure 2:**
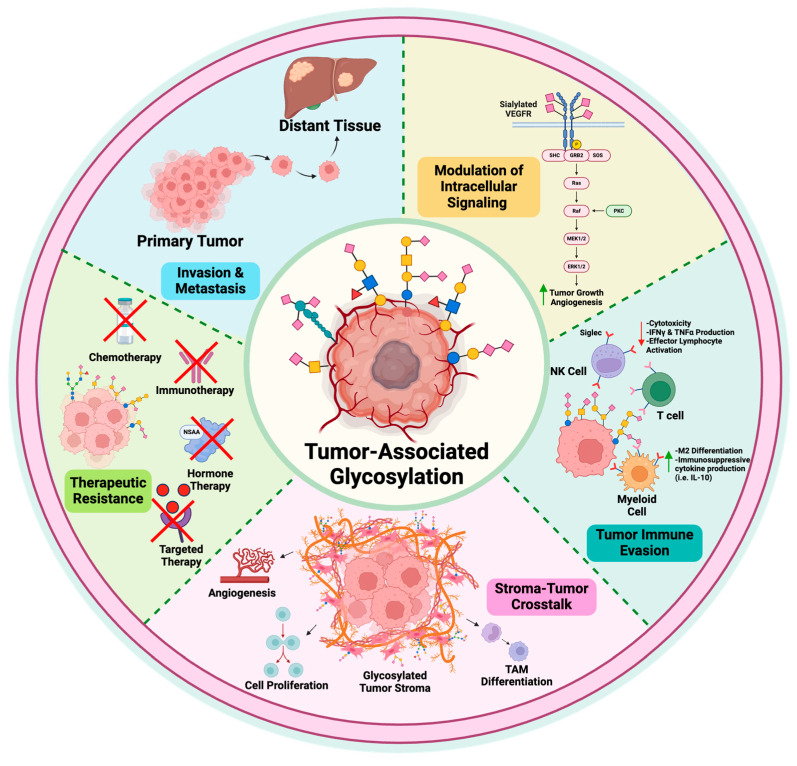
Pathophysiological presentation of tumor-associated glycosylation. TAGs contribute to many of the hallmarks of cancer. As modulators of cell–cell adhesion, TAGs facilitate invasion and metastasis through disrupting the interaction with cell-adhesion molecules such as E-cadherins or integrins. Addition of glycan moieties, such as sialylation, to membrane-bound receptors triggers activation of the MAPK and PI3K-Akt/mTOR pathways. Modulated signaling by these receptors often culminates in increased cell proliferation, resistance to apoptosis, and development of tumor-associated vasculature. Sialic acids enriched on tumor cells are ligands for Siglecs, inhibitory receptors expressed on immune cells. The sialic acid–Siglec interaction diminishes effector function in tumor-fighting NK and T cells and promotes a suppressive phenotype in myeloid cells. Glycan-expressing, stroma-resident CAFs interact with the tumor core to promote angiogenesis and tumor cell proliferation. Increased glycosylation also supports tumor resistance to many different therapeutic modalities through blocking the receptor from the therapeutic or through altering drug efflux into the cell. Created with BioRender.com.

As alluded to in the previous section, TAGs are not only structures enriched in cancer cells, but these modifications can also contribute to disease progression. This section will explore the functional consequences of altered glycosylation in tumor cells. To underscore the significance of these glycan modifications in cancer pathogenesis, these mechanisms have been featured in Figure 2.

### 3.1. Modulation of Signal Transduction

Intracellular signaling enables cells to sense and respond to changes in their environment and plays a key role in determining their fate [42]. In cancer, several hallmarks such as uncontrolled cell growth, resistance to cell death, and increased blood vessel formation can be traced back to disrupted signaling pathways. Changes in glycosylation, particularly at the cell surface, are often upstream contributors to these altered signals [43]. A key site of these changes is membrane-bound receptors, many of which control pathways related to proliferation, survival, and oncogenic signaling. These receptors frequently contain multiple N-glycosylation sites, making them especially susceptible to glycosylation-related changes [5]. Examples of such receptors include FGFR, IGF-1R, VEGFR2, EGFR, and FasR [44,45,46,47,48,49]. Aberrant glycosylation of growth factor receptors like FGFR1/2 and IGF-1R has been linked to enhanced receptor activity, contributing to tumor growth and survival in various cancers. Similarly, sialylation of VEGFR2 has been shown to promote blood vessel formation in vivo. The addition of CA19-9 to EGFR resulted in hyperactivation of the receptor that promoted PDAC tumor progression. Sialylation of EGFR also enhanced ovarian cancer cell proliferation [49]. FasR belongs to the TNF family of death receptors. Accordingly, activation of FasR triggers a caspase cascade resulting in apoptosis. Unlike growth factors, changes in glycosylation of FasR have an inhibitory effect. α-2,6 sialylation of FasR blocked its ability to associate with the Fas-associated death domain (FADD) and initiate the apoptosis signaling cascade [50].

Alterations in glycosylation of cell surface receptors can significantly influence downstream signaling pathways. Although cancer-associated glycoconjugates may differ in their specific structures and functions, many converge downstream through activation of similar signaling pathways. Two of the most well-studied pathways affected by aberrant glycosylation in cancer are the mitogen-activated protein kinase (MAPK) pathway and the PI3K-Akt pathway [5,7,8,9,33]. These pathways are typically activated by receptor tyrosine kinases (RTKs), which include the receptors for most growth factors. Changes in glycosylation can lead to abnormal activation of these receptors. This, in turn, increases phosphorylation of MEK and ERK in the MAPK pathway, as well as activation of mTOR downstream of the PI3K-Akt pathway. Overactivation of these signaling pathways promotes key cancer traits, including enhanced cell growth, survival, and progression through the cell cycle [51,52].

### 3.2. Glycans in Invasion and Metastasis

Tumor metastasis occurs when cancer cells gain the ability to survive and invade tissues beyond the primary tumors. These cells can enter (intravasate) and exit (extravasate) through the lymphatics and circulation, and finally, colonize distant tissue to resume proliferation at the new site [53]. This dynamic nature also allows the metastatic cells to adapt to changing environments encountered during this process [54,55].

In cancer, integrin integrity is often compromised from both structural and functional standpoints. Integrins are transmembrane proteins that comprise non-covalently linked α and β subunits that form a heterodimer [56]. These proteins mediate cell adhesion through cell-ECM interactions and relay signals from the ECM intracellularly [56]. Thus, aberrations in integrin glycosylation have been linked to cell adhesion alterations that promote metastasis [57]. Although α5β1 integrins are established carriers of N-linked glycans, tumor cells exhibit increased branching of these glycans, which reduces adhesion to fibronectin in the ECM [58]. The increased β-1,6 branching was attributed to overexpression of glycosyltransferase GnT-V, which is closely associated with invasion and metastasis. Other glycan modifications have been observed to modulate integrin function. α-2,6 sialylation of β1 integrins increased colon cancer cell migration towards ECM collagen I but not fibronectin [59]. Aberrant O-mannosylation of β1 and β4 integrins increased cell–laminin adhesion and invasion in ovarian cancer [60]. These glycosylation changes likely contribute to metastatic potential by modulating the binding affinity between integrins and ECM components. The fate of these changes, either strengthening or weakening of the interaction, appears to depend on the specific glycan modification or the integrin subunit involved; however, further comprehensive studies are needed to establish a more conclusive understanding.

To accommodate an ever-changing environment, cancer cells often exploit the epithelial-to-mesenchymal transition (EMT) for enhanced cellular plasticity and motility [61]. In non-cancerous cells, EMT is the process in which epithelial cells lose apical-basal polarity and lateral cell–cell junctions and then acquire a spindle-like morphology and display reduced cell–cell adhesion [62]. Recently, TAG, notably sialylation, has been implicated in driving EMT of tumor cells [63]. Sialylation-mediated EMT has been documented in ovarian and pancreatic tumorigenesis [64,65]. This observation is consistent with previous findings linking hypersialylation to the induction of a highly metastatic, cancer stem-like phenotype in multiple tumor types [63,64,65]. In ovarian cancer cells, α-2,3 sialylation has been shown to promote EMT through activation of transforming growth factor-β1 [65]. Similarly, increased α-2,6 sialylation has been associated with the upregulation of EMT-related genes and the induction of pancreatic acinar-to-ductal metaplasia—an early event in pancreatic tumorigenesis accompanied by enhanced tissue invasion and EGFR activation [65]. EGFR appears to play a key role in sialylation-driven EMT and the acquisition of stem-like features in cancer; however, the precise molecular underpinnings linking sialylation to cancer stemness and metastasis remain not fully understood [65,66].

### 3.3. The Role of Glycans and Stromal Dynamics in Tumor Evolution

The fibrotic stroma surrounding many tumors functions as both a physical and therapeutic barrier for the tumor. Tumor stroma typically comprises various forms of collagen, hyaluronic acid, fibronectin, proteoglycans, and cancer-associated fibroblasts (CAFs) [67]. For many malignancies, the stroma acts as a mediator for tumorigenesis and modulation of the cellular landscape of the TME [68]. Although still a relatively unexplored area, the interplay between TAGs and the surrounding stroma is becoming clearer. This relationship appears to contribute to tumor growth and progression, particularly through two major stromal components: CAFs and proteoglycans. CAFs, which are commonly found in the stroma of colorectal and pancreatic tumors, have been shown to express high levels of α-2,3 and α-2,6 sialylated glycans [69,70,71]. These sialylation patterns may influence how CAFs interact with tumor cells and support a pro-tumorigenic environment. In PDAC tumors, the sialic acid residues expressed on CAFs were observed to interact with resident myeloid cells in the TME. This interaction induced differentiation of monocytes to immunosuppressive TAMs, underscoring the role of stroma sialylation in TME [71].

In the presence of cancer cells, fibroblasts within the stroma exhibit increased expression of O-glycans, particularly truncated forms, indicating that tumor–stroma interactions can influence glycosylation patterns. These truncated O-glycans have been shown to regulate the cyclin-dependent kinase 4 (CDK4)–retinoblastoma protein (pRB1) axis in stromal fibroblasts [72]. Since CDK4 and pRB1 are critical regulators of cell cycle progression, alterations in this pathway can lead to unchecked cellular proliferation. These glycosylation-driven changes in stromal cells mirror the aberrant glycosylation events commonly observed in cancer cells, suggesting a shared mechanism contributing to tumor progression.

Tumor–stroma interactions are also influenced by proteoglycans, a class of glycoconjugates composed of one or more glycosaminoglycan (GAG) chains attached to a core protein. In normal tissues, proteoglycans regulate processes such as cell growth, angiogenesis, and extracellular matrix (ECM) homeostasis [73]. However, within the tumor stroma, proteoglycans are often overexpressed or exhibit irregular glycosylation patterns, leading to disruption of these regulatory functions [5]. These changes often culminate in functional consequences, affecting the binding to growth factors, chemokines, and other cell surface receptors that modulate cell migration and survival. For example, in breast cancer, the proteoglycan glypican-1 has been shown to bind α3 chains of type V collagen, a key structural component of the tumor stroma [74]. Loss of this interaction resulted in reduced tumor cell proliferation, highlighting the functional importance of glycan-mediated signaling in tumor–stroma dynamics. Another stromal proteoglycan, versican, is involved in various processes related to vascular development. In cancer, stromal versican has been shown to promote tumor-associated blood vessel formation, supporting the metabolic demands of the growing tumor [75]. These observations are suggestive that abnormal glycosylation of proteoglycans facilitates bidirectional cross-talk between tumor cells and the stroma, ultimately promoting tumor progression.

### 3.4. Sialylation, Inhibitory and Activating Siglecs, and Immune Evasion

A foundational property of the immune system is the ability to distinguish “self” from “non-self.” The addition of glycan moieties to the cell surface is often recognized as “self” structures by the immune system, whereas “non-self” entities possess different glycosylation structures. In the context of cancer, tumor cells are often decorated with similar “self” glycosylation patterns to evade eradication by the immune system. Among the various components of the tumor glycome, the relationship between sialic acids and immune suppression is one of the most well-characterized. Sialic acids serve as ligands for sialic acid-binding immunoglobulin-like lectins (Siglecs), a family of receptors predominantly expressed on the surface of immune cells. In humans, there are 14 functional Siglec receptors, and several Siglec homologues are also expressed in mice [76]. Siglecs comprise both ITIM-bearing inhibitory receptors and DAP12-coupled activating receptors; in tumors, hypersialylated ligands generally skew signaling toward inhibition (reduced cytotoxicity, increased TAM polarization), although cell-type and context determine whether inhibitory or activating pathways dominate [76]. Most Siglecs display a specificity towards α-2,3 and α-2,6 sialic acids, which are highly expressed on tumor cells, as opposed to α-2,8 linkages [8,33]. Accordingly, the sialic acid–Siglec axis is associated with poor patient outcomes, such that high expression of these moieties in ovarian and pancreatic malignancies has been linked with poor survival and resistance to conventional anticancer therapeutics [77,78]. Some of the strongest facilitators of durable antitumor immunity, T cells and natural killer (NK) cells, are targets of suppression from the sialic acid–Siglec axis. In human PDAC, high expression of sialylation-related genes correlated with reduced tumor-infiltrating CD8+ T cells and increased regulatory T cell populations, characteristic of an immunosuppressive TME [61]. Accordingly, blocking the sialic acid and Siglecs-7/9 interaction inhibited prostate tumor growth, as well as increasing CD4+ and CD8+ T cell infiltration [79]. In NK cells, cytotoxicity and IFN-γ production were enhanced following desialylation of tumor cells, reducing the interaction with Siglecs-7/9 [80]. Tumor sialic acids also target Siglecs expressed on myeloid cells to promote tumor immunosuppression. In addition to T cell and NK cells, Siglec-7 and Siglec-9 also contribute to the differentiation of intratumoral monocytes into tumor-associated macrophages (TAMs), which support a suppressive TME [81]. This process was largely driven by the interaction between α-2,3 sialic acids and Siglec-9, leading to the upregulation of immunosuppressive TAM-associated markers such as CD163, CD206, and IL-10.

Beyond ITIM-bearing inhibitory Siglecs (e.g., Siglec-7/9), several activating Siglecs signal via DAP12 to promote immune cell activation. Tumor sialoglycans can bias this balance by preferentially engaging inhibitory Siglecs on myeloid and NK/T cells, while limited engagement of activating Siglecs constrains anti-tumor function. Accordingly, the net outcome of sialic acid–Siglec interactions in the TME reflects the relative expression of ligands, inhibitory receptors, and activating counterparts.

Regarding signaling directionality of sialic acid–Siglec interactions, it is generally accepted that the Siglec is the “receiver” of this interaction, and that this signal is transduced in only one direction [33,76]. A recent report suggested the signaling from this may be bidirectional, in which both the sialic acid-expressing cell and the Siglec-expressing cell are the signal “receivers.” The interaction between Siglec-7 and sialylated T antigen resulted in suppressed cytotoxicity in NK cells, a more canonical mechanism. In addition, this interaction transduced signals to the cancer cell, increasing migration and invasiveness. Though we have some understanding of how tumor-associated glycosylation shapes the immune landscape of the TME, understanding the extent to which the TME shapes the fate of the tumor glycome certainly warrants more consideration.

### 3.5. Therapeutic Resistance

With many therapeutic modalities for cancer, treatments often accentuate tumor cell stresses and implement rapid stress mitigation responses to sustain cell viability [82]. Survival and propagation of these cells give rise to an even more aggressive, treatment-resistant population of tumor cells [83]. As the molecular and cellular mechanisms underlying therapeutic resistance become better understood, tumor-associated glycans have emerged as potential contributors to therapeutic shortcomings. Among the various glycan alterations implicated in therapeutic resistance, changes in N-glycans are the most documented and are used in targeted therapies. This may be attributed to the physical bulk of glycan structures on therapeutic targets, which can hinder drug binding or receptor accessibility. Analysis of the glycan landscape of prostate tumors from patients receiving initial doses of hormonal therapy, as well as those in remission, revealed a shift in N-glycan composition. Specifically, biantennary structures were increased while more complex tri- and tetraantennary N-glycans were less abundant. However, tri- and teraantennary N-glycans were more abundant in tumors that had acquired resistance to hormonal therapy, underscoring the importance of glycan structures in therapeutic resistance [84]. N-glycan branching also appears to impact monoclonal antibody-based therapies [18]. Branched N-glycans present on tumor-associated vasculature reduced the sensitivity of anti-VEGF therapies, as a result of the glycan antennae functioning as a structural hindrance, preventing the therapeutic from accessing its binding site [18]. Altered glycosylation of immune checkpoint proteins PD-L1/2 also have been recognized in modulating therapeutic efficacy. Removal of N-linked glycans on PD-L1 enhanced protein detection, thus improving the therapeutic efficacy of anti-PD-1/PD-L1 blockade [18]. Fucosylation of PD-L2 facilitated interaction with EGFR and activation of the STAT3 signaling pathway. This interaction resulted in reduced affinity for EGFR inhibitor cetuximab as well as diminishing its therapeutic efficacy [85]. These studies suggest that responders to immune checkpoint inhibitors (ICIs) may display a different glycosylation signature on these targets than non-responders. Thus, identification of ICI target glycosylation patterns has the potential to better predict response to these therapies.

## 4. Therapeutic Targeting of Tumor-Associated Glycans

A basis for therapeutic targeting of glycans in cancer is that glycosylation alterations are integral in stratifying malignant cell populations as well as supporting many of the capabilities acquired by tumor cells. Advances in the understanding of tumor glycobiology have revealed that many aberrant glycan structures are either absent or expressed at very low levels on non-malignant cells, making them attractive targets for more selective therapeutic approaches. Given the limitations of many current cancer treatments, which lack specificity and have many associated toxicities, targeting tumor-associated glycans offers a potential strategy to improve therapeutic precision and reduce adverse effects. The unique advantages of glycan-targeting therapies have even led to further investigation with in-human clinicals, several of which are featured in Table 1. In addition, this section highlights several emergent glycan-targeting strategies that hold significant promise for further enhancing cancer care.

Despite promising preclinical activity, several glycan-targeting strategies have demonstrated limited clinical efficacy or dose-limiting toxicity, largely due to the widespread expression of glycans on normal tissues and insufficient tumor specificity. Future success will likely depend on improved target selectivity, context-restricted delivery approaches, and rational combination with immunotherapies or standard treatments to enhance efficacy while minimizing off-tumor effects.

### 4.1. Antibody-Based Therapeutics

Therapeutic monoclonal antibodies (mAbs) are regarded as one of the most effective treatment strategies for many cancer types over the past three decades. Their clinical utility has expanded considerably due to their ability to bind target antigens with high specificity and to induce long-lasting antitumor immunity [108]. Upon interaction with its target, mAbs initiate cytotoxicity, neutralization, and/or additional antitumor immune responses [109]. Therapeutic mAbs have been constructed to interfere with glycan-receptor interactions, in addition to the development of more canonical mAbs that are raised against tumor-associated glycans. In a murine model of glioblastoma, mAb blockade of inhibitor receptor Siglec-E (the murine homolog of Siglec-9) disrupted the interaction between immune cell-expressed Siglecs and tumoral sialic acids, providing significant survival benefit [110]. Importantly, deletion of Siglec-E enhanced antigen presentation and CD86 expression, generating an intratumor milieu that is sensitive to other mAb therapies, including PD-1 and CD47 mAb blockade. Moreover, blocking of Siglecs-7/9 with mAbs, thereby interfering with the Siglec and sialyl Lewis^A^ interaction, reduced tumor burden and lung metastasis in mice [111]. Building upon this approach, mAbs against tumor-associated glycans themselves are also a promising strategy in mitigating tumor progression. 4C8, a mAb that binds Tn-glycosylated CD44v6, demonstrated robust selective reactivity towards tumor cells and cancerous tissue [112].

For many therapeutic mAbs, glycosylation of the target antigen can obstruct effective binding by masking the epitope, thereby limiting therapeutic efficacy [113,114,115]. Additionally, variations in glycan structures presenton target molecules can further influence mAb effectiveness in cancer treatment. To address this limitation, enzyme–antibody constructs are being developed to enzymatically remove glycans from the mAb target. E-301 is an example of such a construct, consisting of two sialidase domains that are fused to the C-terminal Fc region of trastuzumab [116]. This fusion protein selectively removes sialic acids from cells within the TME. Treatment with E-301 drastically suppressed tumor growth, and enhanced Ki67 and granzyme B expression from CD8+ T cells. In addition, E-301 shifted TAMs from an immunosuppressive M2 phenotype to a more pro-inflammatory M1 phenotype [116].

### 4.2. CAR T Cell Therapy and BiTEs

Chimeric antigen receptor (CAR) T cells are engineered with CARs, synthetic receptor proteins that allow effector T cells to target a specific antigen. Upon recognition of this antigen, CAR T cells are robustly activated and direct potent cytotoxicity towards the tumor cells expressing the antigen [117]. The precision and high durability of the T cell-mediated antitumor immunity of CAR T cell therapies have provided remarkable clinical responses for hematological malignancies [117,118]. However, similar therapeutic efficacy has yet to be demonstrated with the treatment of solid tumors [118]. Although this limited efficacy of CAR T cell therapies is recognized to be multifactorial, TAG has been identified as a key contributor to the therapeutic shortcomings [14]. Addressing the limited therapeutic efficacy at its source, glycan-targeting CAR T cell therapies are currently being explored for the treatment of solid tumors. CAR T cells engineered to target the TF antigen have demonstrated tumoricidal effects against lung and pancreatic adenocarcinoma cells. Upon recognition of the TF antigen, these CAR T cells exhibited elevated expression of the activation marker CD69 and increased secretion of IFN-γ, indicating robust T cell activation and effector function [119]. TF is an ideal target for CAR T cell therapy as it is almost exclusively expressed in an unmasked form on tumor cells [120]. Importantly, lectin-expressing CAR T cells provide therapeutic targeting of glycans with even greater stringency. Lectin-CAR T cells comprising the Shiga toxin B-subunit lectin derived from *Shigella dysenteriae* (Shiga-CAR), successfully utilized as a “guide” for the CAR T cells to target glycosphingolipid Gb3, a structure heavily expressed in many tumor types [121]. In the presence of tumor cells, Shiga-CAR demonstrated target-specific cytotoxicity against Gb3-expressing colorectal and breast cancer cells, through inducing direct tumor cell lysis and release of effector cytokines IFN-γ, TNF-α, and granzyme B [121]. Lectin-CAR targeting has also been effective in vivo against primary tumors, and the desmoplastic stroma surrounds the tumor. Targeting tumor stroma can drastically improve drug delivery, immune cell infiltration, and survival outcomes [122,123,124]. CAR T cells expressing a modified banana lectin (H84T-CAR) targeted highly mannosylated glycoconjugates, which are associated with tumor progression. H84T-CAR treatment significantly reduced PDAC tumor growth, as well as disrupting the highly fibrotic tumor-associated stroma, through targeting highly mannosylated, stroma-producing pancreatic stellate cells [31].

Emergent T cell targeting immunotherapies, such as bispecific T cell engagers (BiTEs), are also utilizing TAG targets for improved therapeutic efficacy and precision. BiTEs consist of two single-chain variable fragments: one directed against CD3 on T cells and the other targeting a tumor-associated antigen or immunomodulatory molecule, thereby facilitating T cell-mediated cytotoxicity against tumor cells expressing the target antigen [125]. While BiTEs have been highly effective in the treatment of acute lymphoblastic leukemia, therapeutic success in solid tumors has thus far been limited [126]. A new but promising strategy to improve BiTE cytotoxicity in solid tumors is enhancing the accessibility of the tumor cell target at the BiTE-induced interface between the tumor cell and T cell. A fusion protein comprising a HER2-targeting BiTE and a sialidase has been utilized to potentiate BiTE cytotoxicity through selective tumor cell desialylation [127]. improved immunological synapse formation between T cells and HER2+ target cells, resulting in enhanced T cell activation and cytotoxicity, as measured by CD69 expression and IFN-γ and TNF-α release. The HER2 BiTE sialidase exhibited stronger tumor control over parental BiTEs when used in a HER2+ xenograft tumor model. Building upon this platform, EGFR-targeting and CD19-targeting BiTE-sialidase fusion proteins were developed for the treatment of melanoma and leukemia, respectively. Both BiTE sialidase fusion proteins outperformed the parental BiTE, underscoring the impact of the glycocalyx on the immune and tumor cell engagement required for cell-based therapies such as BiTEs [127].

### 4.3. Glycan Degradation and Inhibition

Altered glycosylation patterns on tumor cells support cancer pathogenesis from multiple fronts. In addition to the qualities discussed in the previous section, the accumulated bulk of glycan additions can function as a physical hindrance to targeted therapies and antitumor immune cells, effectively preventing access to the tumor. Indeed, the thickness of the tumor glycocalyx has been inversely correlated with NK cell cytotoxicity [128]. A strategy that is actively being explored to sensitize tumors to targeted therapies is through the removal of glycan moieties from the cell surface, primarily through the addition of glycan-degrading agents. Tumoral sialic acids have been the primary target for therapeutic glycan removal, largely due to their localization at the end of many glycan structures [33]. Desialylation of tumor cells alone has been proven effective in inducing antitumor immunity in ovarian cancer. A sialidase-treated whole tumor cell vaccine enhanced DC activation and T cell cytotoxicity against A2780 and OVCAR3 cells [74,129]. In addition, sialic acid degradation has been implemented in interrupting the sialic acid–Siglec interaction. To enhance the potency of mesothelin-targeting iPSC-derived CAR macrophages (CAR-iMacs), a sialidase was added as a combination therapy [130]. Targeted removal of sialic acids attenuated the “don’t eat me signal” from Siglec-5 and Siglec-10, resulting in enhanced CAR-iMac polarization and activation. Consequently, the CAR-iMac activation shifted the TME from a pro-tumor, immune evasive state to an anti-tumor, immune-inflamed state [130]. Glycan degradation-mediated interruption of the sialic acid–Siglec interaction has also been investigated in antibody-based therapeutics. T-Sia-2 is an antibody-sialidase conjugate, targeting HER2. Both in vitro and in vivo examination of T-Sia-2 demonstrated significant specificity towards sialylated HER2 [34]. Further, treatment with T-Sia-2 blocked interaction with Siglec-E expressed on myeloid cells, potentiating antitumor immunity [117]. Another important consideration with therapeutic sialic acid degradation is that sialic acids support tumor progression through both tumor extrinsic and intrinsic factors [18,19,20,21,22]. Thus, degradation of sialic acids also has the advantage of directly affecting the tumor cell. Direct treatment of bladder cancer cells with sialidase Neu1 reduced cell proliferation and increased tumor cell apoptosis [131]. Neu1 treatment reduced cell growth and apoptosis evasion through downregulating the PI3k-Akt signaling pathway, which is a key mediator in bladder cancer growth [131].

Although less thoroughly investigated, the targeted degradation or inhibition of other tumoral glycan structures have also demonstrated promising results in limiting tumor progression. To enhance the antitumor activity of CAR NK cells, the cells were engineered to express the mucinase StcE, an enzyme derived from *Escherichia coli* that specifically targets the MUC1 glycoprotein [128]. Mucins such as MUC1 are overexpressed on the surface of cancer cells, and the long chains confer protection against cytotoxicity from effector immune cells [132]. Thus, CAR NK cells equipped with StcE were superior in tumoricidal ability and accessing the CAR target, HER2 [116]. Inhibition of aberrant glycan synthesis is another promising therapeutic approach. Kifunesine is an α-mannosidase inhibitor, which prevents the formation of branched or complex N-glycans. In ovarian cancer cells, kifunesine treatment reduced tumor cell aggregate and spheroid formation, a key event in ovarian cancer pathogenesis that supports chemoresistance and metastasis of tumor cells in ascites [133]. Therefore, more investigations into kifunesine’s antimetastatic properties is certainly warranted.

### 4.4. Small Molecule Therapeutics

Small molecule therapies provide the benefit of greater tumor accessibility while minimizing adverse toxicities that often accompany the usage of many macromolecule-based therapeutics [134,135]. Advancements in our understanding of TAG has sparked interest in the development of glycan-targeting small molecule therapies, as these glycan modifications play a critical role in cancer pathogenesis [5,7,8,9]. By targeting these specific glycan structures, small molecules can potentially disrupt the molecular mechanisms driving tumor progression. While this is a highly emergent portion of small molecule therapeutics, promising findings have been observed through targeting tumor-associated glycans from a biosynthetic standpoint. 2-Deoxy-d-glucose (2DG) is a glucose/mannose analog that inhibits the formation of branched N-glycans [136]. Reduced N-glycan synthesis from 2DG exposure has been shown to sensitize ovarian tumors to anti-PD-L1 blockade [137]. Additionally, 2DG enhanced CAR T cell-mediated cytotoxicity against pancreatic tumors by promoting effective immunological synapse formation between the CAR T cell and tumor cell [138]. P-SiaFNEtoc, a sialyltransferase inhibitor, specifically blocks the formation of α-2,6 sialic acid linkages [139]. Treatment with P-SiaFNEtoc resulted in reduced primary tumor growth in the TRAMP-C2 prostate cancer model and suppressed the development of skeletal metastases and improved tumor outcomes [38].

### 4.5. Glycomimetics

Glycomimetic therapies are a burgeoning treatment modality centered on manipulating the glycosylation patterns on tumor cells. Targeting the glycosylation processes with synthetic molecules, known as glycomimetics, these molecules aim to inhibit cancer cell adhesion, metastasis, and the ability of tumors to evade immune detection [5]. The structural diversity of glycans has lent itself to the development of highly diverse glycomimetic molecules. PAMAM-GD2 and PAMAM-GD3 are glycomimetic cancer vaccines comprising ganglioside GD2 or GD3 analogs that are conjugated to a tetrameric polyamidoamine scaffold [140]. GD2 and GD3 are suitable targets for glycomimetic therapies, as they are considered tumor-biomarker gangliosides for a number of tumor types [141]. Both PAMAM-GD2 and PAMAM-GD3 were effective in establishing both humoral and cellular immunity against tumor-expressed gangliosides [140]. Notably, both vaccines generated CD8+ T cells and γδ T cell receptor-expressing lymphocytes that were effective in mitigating primary tumor and metastatic progression of GD2+ or GD3+ tumors [127]. CLEC10A is a lectin-like receptor that is specific for terminal GalNAc residues with many immunostimulatory properties upon activation; however, natural ligands of CLEC10A possess low binding affinity. Screening of a phage display library with a GalNAc lectin identified sv6D, a 6-peptide GalNAc mimetic with high binding affinity for CLEC10A [142]. sv6D was effective in expanding cytotoxic T cell, NK cell, and NKT cell populations in the peritoneum of mice, and rendering ovarian cancer tumors sensitive to anti-PD-1 blockade [142]. As a mediator of ECM-tumor cell interactions, GAG mimetics are also under investigation. AlgSulf is a heparin GAG mimetic that preferentially inhibited the proliferation and invasion of tumorigenic breast epithelial cells in 2D, 3D, and coculture models, while preserving the differentiation and acinar structure characteristic of non-tumorigenic mammary cells [143]. Importantly, the antitumor properties of AlgSulf surpassed that of native heparin, which may be attributed to altered growth factor interactions. Also utilizing GAGs, heparan sulfate GAG mimetic KI-105 effectively impeded the migration and invasion of human fibrosarcoma HT1080 cells. The compound appeared to enhance tumor cell adhesion, a characteristic of cells with less propensity to invade or metastasize. Altered adhesion may be attributed to the increasing cell–surface GAG levels and stabilizing focal adhesions, suggesting involvement of both heparanase inhibition and modulation of Cdc42 signaling [144].

## 5. Conclusions

Tumor-associated glycosylation is increasingly recognized as a critical determinant in cancer pathogenesis, shaping multiple facets of tumor biology, including cell proliferation, immune evasion, metastatic dissemination, and therapeutic resistance. Alterations in glycosylation patterns—marked by changes in glycan structure, branching, and composition—are largely driven by dysregulated expression of glycosyltransferases, glycosidases, and other enzymes involved in glycan biosynthesis and remodeling. These aberrant modifications influence key cellular processes such as receptor signaling, cell–cell and cell–matrix interactions, and communication within the tumor microenvironment, collectively supporting the acquisition of hallmark traits of malignancy.

One of the most prominent consequences of dysregulated glycosylation is its role in subverting immune surveillance. Overexpression of sialylated glycans or aberrant fucosylation can create a glycan shield on the tumor cell surface, reducing recognition by immune cells and promoting immunosuppression. These findings underscore the therapeutic potential of targeting glycosylation pathways to enhance anti-tumor immune responses and restore effective immune surveillance. In addition to immune modulation, glycosylation plays a significant role in promoting tumor invasion and metastasis. Altered glycosylation of cell surface receptors and ECM components facilitates enhanced adhesion, motility, and tissue infiltration, enabling tumor cells to disseminate and colonize distant sites. A deeper understanding of these glycan-mediated interactions may offer new opportunities for therapeutic intervention aimed at limiting metastatic progression. Despite the growing body of knowledge, the clinical translation of glycosylation-based insights faces several challenges. The structural complexity and heterogeneity of glycan modifications across different tumor types hinder the development of broadly applicable therapeutic strategies. Moreover, the absence of sensitive and specific biomarkers for detecting aberrant glycosylation in early-stage disease remains a significant limitation in its application for cancer diagnostics and personalized medicine.

In summary, aberrant glycosylation is a key contributor to tumor development and progression, influencing immune escape, signaling dynamics, and metastatic behavior. Future investigative efforts should aim to delineate the context-dependent roles of specific glycan alterations in various cancer types, identify reliable biomarkers for early detection, and design targeted therapies that either reverse or exploit these glycosylation changes for targeting with other available therapeutics. Indeed, as our understanding of tumor-associated glycosylation deepens, implementing this knowledge into drug development is poised to generate a new class of targeted cancer therapies. Leveraging the distinct structural and functional properties of tumor-associated glycans, significant advancements are being made to develop therapies that cater to these features, potentially offering greater potency and selectivity than conventional treatment modalities. Looking ahead, continued innovation in this space offers great promise to expand the therapeutic arsenal beyond established targets and conventional therapeutics, and enable the development of precision medicines that address the complexity and heterogeneity of cancer at the glycan level.

While many associations between aberrant glycosylation and tumor progression are supported by convergent evidence across cancer types and model systems, the precise causal mechanisms and context-specific dependencies remain incompletely defined and, in some settings, are supported primarily by correlative or preclinical data. Key unanswered questions include how intratumoral and interpatient heterogeneity shapes the tumor glycome, how glycosylation dynamically evolves during therapy, and how glycan-targeted agents can be optimally integrated with immunotherapies to achieve durable clinical benefit.

## Figures and Tables

**Table 1:** Therapeutic glycan targeting and modulation in clinical trials.

Candidate Drug	Class	Description/Mechanism of Action	Cancer Type	Phase	Status/Results (If Applicable)	Trial Number	Reference
GD2-CAR T	CAR T Therapy	CAR T cell targeting of ganglioside GD2	H3K27-mutant diffuse intrinsic pontine glioma, spinal diffuse midline glioma	I	Recruiting Seven of eleven patients enrolled in the trial exhibited reduced tumor size, four of which reduction was >50% original tumor size. Responders to therapy exhibited elevated IL-2 in plasma.	NCT04196413	[86,87]
NEO-201	Monoclonal Antibody	IgG1 mAb that recognizes a core-1 O-glycan–dependent epitope preferentially displayed on tumor cells; mediates ADCC	Solid Tumors	I	Recruiting for the expansion phase Safe and well-tolerated at MTD of 1.5 mg/kg. Four patients experienced stable disease with therapy.	NCT03476681	[88]
Naxitamab	Monoclonal Antibody	Targets ganglioside GD2, induces antibody-dependent cellular toxicity and complement-dependent cytotoxicity	Relapsed/refractory neuroblastoma	II	Recruiting The overall response rate of 50% for the whole study. Complete and partial responses in 38% and12% of patients, respectively. Manageable adverse events.	NCT03363373	[89]
SGN-2FF	Small Molecule	Inhibitor of glycoprotein fucosylation, L-fucose analog	Advanced solid tumors	I	Terminated One patient achieved a complete response, and 36% of patients had stable disease. Thromboembolic events occurred and precluded further study.	NCT02952989	[90]
Uproleselan	Glycomimetic	E-Selectin antagonist, disrupts cell adhesion and survival	Relapsed/Refractory acute myeloid leukemia	I/II	Completed Safe and well-tolerated from 5 to 20 mg/kg. 41% remission rate and median overall survival of 8.8 months at 10 mg/kg, and twice daily dosage.	NCT02306291	[91]
DS-8895a	Monoclonal Antibody	Targets EPAH2, afucosylated mAb to enhance ADCC	Advanced solid tumors	I	Completed Generally safe and well tolerated, 8.1% of patients experienced ≥3 AEs. 6.7% of patients experienced a partial response, 33.3% experienced stable disease. Dose-dependent activation of NK cells was also observed.	NCT02004717	[92]
Tecemotide	Cancer Vaccine	Comprises a recurring sequence found in glycoprotein MUC-1, elicits an immune response against MUC-1	Breast cancer	II	Completed Tecemotide did not provide any therapeutic benefit as an adjuvant therapy to hormonal therapy or chemotherapy. No additional adverse toxicities were observed when added to standard of care therapies.	2011-004822-85 (EU)	[93]
OPT-821	Cancer Vaccine	Comprises gangliosides GD2 and GD3 conjugated to protein carrier KLH. Stimulates the immune system against gangliosides GD2 and GD3	High-risk neuroblastoma	II	Active, not recruiting Therapy was well-tolerated, no > grade 3 toxicities. A higher anti-GD2 antibody titer from the vaccine therapy was associated with improved survival.	NCT00911560	[94]
MUC1 Peptide Vaccine	Cancer Vaccine	Comprises 100 amino acids of the MUC1 sequence and TLR3 agonist Poly ICLC, elicits an immune response against MUC-1	Recurrent colorectal adenoma	II	Active, not recruiting Therapy increased anti-MUC-1-IgG2 40-fold. No change in adenoma recurrence compared to placebo. No change in serious adverse events occurred between the vaccine and placebo groups.	NCT02134925	[95]
GD2BATs	Bispecific Antibody Armed T cells	Anti-CD3, anti-GD2 targeting, bispecific antibody armed T cells, that potently eradicate ganglioside GD2-expressing tumor cells	Neuroblastoma and osteosarcoma	I/II	Status unknown No dose-limiting toxicities observed in both segments. In Phase I, one patient had a complete bone marrow response. In Phase II, 10% of patients experienced a partial response, and 50% had stable disease. Over 50% Phase II patients exhibited enhanced GD2+ immune responses post-treatment.	NCT02173093	[96]
GD2-CART01	CAR T Therapy	CAR T cell targeting of ganglioside GD2, GD2-CART01 cells are engineered with an inducible caspase 9 gene to kill the cells if therapy is associated with adverse toxicities	Relapsed/refractory neuroblastoma	I/II	Active, not recruiting Treatment was safe, and adverse toxicities were controlled by the activation of the suicide gene. 63% of patients responded to therapy, 33% had a complete response, and 30% experienced a partial response.	NCT03373097	[97]
JNJ-67571244	Bispecific Antibody	CD33 (Siglec-3) x CD3 targeting bispecific antibody, that bridges T cell and cancer cell interaction, facilitating clearance of CD33+ cancer cells	Relapsed/refractory acute myeloid leukemia and myelodysplastic syndrome	I	Completed No overall response greater than stable disease was observed. All patients in the study experienced ≥1 treatment-emergent adverse event.	NCT03915379	[98]
Anti-CD19 CAR T + Anti-CD22 CAR T	Sequential CAR T Therapy	Staggered infusion of CAR T cells targeting CD19, followed by CAR T cells targeting CD22 (Siglec-2) to potentiate CAR T cell-mediated clearance of malignant cells	Relapsed/Refractory B cell acute lymphocytic leukemia	II	Terminated Therapy was well tolerated and had manageable treatment-related toxicities. 80% of patients had leukemia-free survival of one year, and only three patients had relapses after treatment.	NCT04340154	[99]
Anti-CD19/CD22 CAR T cells	Bispecific CAR T cells	CAR T cells that target both CD19 and CD22 (Siglec-2) simultaneously, to mitigate antigen loss-associated relapse	Adult B cell acute lymphocytic leukemia with measurable residual disease	I	Completed Treatment was generally well-tolerated; however, several ≥ grade 3 hematologic-related adverse events also occurred. 100% of patients with measurable residual disease responded to therapy, but the median relapse-free survival was not met.	NCT03919526	[100]
Ad-sig-hMUC1/ecdCD40L	Cancer Vaccine	Recombinant adenovirus-based vaccine comprising MUC1 antigen fused to the ECD of CD40L, to elicit MUC1-targeting antitumor responses	Advanced adenocarcinoma	I	Status Unknown Therapy was safe and well-tolerated, no ≥ grade 3 drug-related adverse toxicities were reported. No partial or complete responses were observed; however, 48% and 33% of patients had stable or progressive disease, respectively. Therapy increased cytotoxic CD8+ T cells and B cells in patients with stable disease.	NCT02140996	[101]
Gatipotuzumab	Monoclonal Antibody	Targets MUC1, induces antibody-dependent cellular toxicity and phagocytosis	Refractory solid tumors	Ib	Completed Therapy was well-tolerated, and no adverse toxicities were observed when combined with an anti-EGFR mAb. Partial responses were observed in colorectal cancer patients.	NCT03360734	[102]
Inotuzumab Ozogamicin	Antibody-Drug Conjugate	Comprises an anti-CD22 (Siglec-2) antibody conjugated to a calicheamicin antitumor antibiotic. Delivers cytotoxic agent to malignant cells upon internalization from binding antibody target.	B-cell precursor acute lymphoblastic leukemia	II	Active, not recruiting Therapy was generally well-tolerated after the second induction, with a few adverse events being neutropenia and thrombocytopenia. After the second treatment induction, 90% of patients experienced complete remission, and 80% had measurable residual disease. One-year relapse-free survival was 66%.	NCT03249870	[103]
SC-DARIC33	DARIC T cells	Drug-regulated (rapamycin) activation and cytotoxicity towards CD33+ (Siglec-3) tumor cells	Acute myeloid leukemia	I	Recruiting Therapy exhibited preferential migration towards malignant tissue (chloroma) and increased expression of T cell activation markers following rapamycin administration.	NCT05105152	[104]
Belapectin	Polysaccharide	Galectin antagonist, targets extracellular galectin-1 and galectin-3	Metastatic melanoma, head and neck squamous cell carcinoma	I	Completed Therapy was well tolerated in combination with pembrolizumab, no grade 4 toxicities were observed. A total of 50% of MM patients and 33% HNSCC patients in the study experienced an objective response to therapy.	NCT02575404	[105]
BI 836858	Monoclonal Antibody	Targets CD33 (Siglec-3), initiates ADCC through NK cell targeting	Acute myeloid leukemia	I/II	Completed Therapy exhibited a manageable tolerability profile. A total of 2% of patients had partial remission, 32.7% had stable disease following therapy combined with decitabine.	NCT02632721	[106]
CD19-22.BB.z	Bispecific CAR T cells	CAR T cells that target both CD19 and CD22 (Siglec-2) simultaneously, to mitigate antigen loss-associated relapse	Recurrent/Refractory B-cell malignancies	I	Active, not recruiting No dose-limiting toxicities were observed, and all CRS and neurotoxicity-related events were resolved. 88% of B-ALL patients exhibited minimal-residual disease, complete remission. Relapses occurred in 50% of patients with B-ALL and 29% of LBCL patients.	NCT03233854	[107]

## Data Availability

No new data were created or analyzed in this study.
